# An Explainable Artificial Intelligence Model Proposed for the Prediction of Myalgic Encephalomyelitis/Chronic Fatigue Syndrome and the Identification of Distinctive Metabolites

**DOI:** 10.3390/diagnostics13233495

**Published:** 2023-11-21

**Authors:** Fatma Hilal Yagin, Abedalrhman Alkhateeb, Ali Raza, Nagwan Abdel Samee, Noha F. Mahmoud, Cemil Colak, Burak Yagin

**Affiliations:** 1 Department of Biostatistics and Medical Informatics, Faculty of Medicine, Inonu University, Malatya 44280, Türkiye; cemil.colak@inonu.edu.tr; 2 Computer Science Department, Lakehead University, Thunder Bay, ON P7B 5E1, Canada; aalkhate@lakeheadu.ca; 3 Institute of Computer Science, Khwaja Fareed University of Engineering and Information Technology, Rahim Yar Khan 64200, Pakistan; ali.raza.scholarly@gmail.com; 4 Department of Information Technology, College of Computer and Information Sciences, Princess Nourah bint Abdulrahman University, P.O. Box 84428, Riyadh 11671, Saudi Arabia; nmabdelsamee@pnu.edu.sa; 5 Rehabilitation Sciences Department, Health and Rehabilitation Sciences College, Princess Nourah bint Abdulrahman University, P.O. Box 84428, Riyadh 11671, Saudi Arabia; nfmahmoud@pnu.edu.sa

**Keywords:** explainable artificial intelligence, myalgic encephalomyelitis/chronic fatigue syndrome, metabolomics data, clinical classification

## Abstract

Background: Myalgic encephalomyelitis/chronic fatigue syndrome (ME/CFS) is a complex and debilitating illness with a significant global prevalence, affecting over 65 million individuals. It affects various systems, including the immune, neurological, gastrointestinal, and circulatory systems. Studies have shown abnormalities in immune cell types, increased inflammatory cytokines, and brain abnormalities. Further research is needed to identify consistent biomarkers and develop targeted therapies. This study uses explainable artificial intelligence and machine learning techniques to identify discriminative metabolites for ME/CFS. Material and Methods: The model investigates a metabolomics dataset of CFS patients and healthy controls, including 26 healthy controls and 26 ME/CFS patients aged 22–72. The dataset encapsulated 768 metabolites into nine metabolic super-pathways: amino acids, carbohydrates, cofactors, vitamins, energy, lipids, nucleotides, peptides, and xenobiotics. Random forest methods together with other classifiers were applied to the data to classify individuals as ME/CFS patients and healthy individuals. The classification learning algorithms’ performance in the validation step was evaluated using a variety of methods, including the traditional hold-out validation method, as well as the more modern cross-validation and bootstrap methods. Explainable artificial intelligence approaches were applied to clinically explain the optimum model’s prediction decisions. Results: The metabolomics of C-glycosyltryptophan, oleoylcholine, cortisone, and 3-hydroxydecanoate were determined to be crucial for ME/CFS diagnosis. The random forest model outperformed the other classifiers in ME/CFS prediction using the 1000-iteration bootstrapping method, achieving 98% accuracy, precision, recall, F1 score, 0.01 Brier score, and 99% AUC. According to the obtained results, the bootstrap validation approach demonstrated the highest classification outcomes. Conclusion: The proposed model accurately classifies ME/CFS patients based on the selected biomarker candidate metabolites. It offers a clear interpretation of risk estimation for ME/CFS, aiding physicians in comprehending the significance of key metabolomic features within the model.

## 1. Introduction

Myalgic encephalomyelitis/chronic fatigue syndrome (ME/CFS) is a complex and debilitating disease. It may come with broad heterogeneity and common symptoms, including severe fatigue, post-exertional malaise (PEM), restless sleep, cognitive impairment, and orthostatic intolerance [1]. The prevalence of ME/CFS is significant, with more than 65 million suffering individuals worldwide, indicating the significant impact of the disease on a global scale [2]. In addition, the true prevalence of the disease is difficult to determine due to factors such as underdiagnoses and misdiagnoses [3]. Although ME/CFS has been observed to be diagnosed more frequently in women, it is not a female-specific condition, and approximately 35–40% of patients with ME/CFS are male [4]. The reasons behind the higher prevalence in women are not fully understood [4,5] and may be influenced by a variety of factors such as hormonal differences, genetic predisposition, and social and cultural factors.

Dysfunctions of various systems, including the immune, neurological, gastrointestinal, and circulatory systems, have been reported in individuals with ME/CFS [6,7,8,9]. Studies focusing on the immune system have revealed abnormalities in various immune cell types among ME/CFS patients, suggesting that the disease is an immune disorder [6,7]. Increased levels of inflammatory cytokines were also observed in the plasma of ME/CFS patients compared with healthy controls, indicating an increased inflammatory response [10].

Neuroimaging studies have identified abnormalities in the brains of ME/CFS patients, including changes in brain structure and function. These findings add to the understanding of cognitive impairment and other neurological symptoms experienced by individuals with ME/CFS [11]. Digestive problems are common among ME/CFS patients, with a significant proportion reporting symptoms consistent with irritable bowel syndrome (IBS). This suggests that the gastrointestinal tract plays a potential role in the pathophysiology of the disease [12,13].

The circulatory system plays a very important role in providing essential compounds and removing metabolic wastes from various organs [14]. Several studies have been conducted to characterize the blood metabolome of ME/CFS patients to gain insight into the underlying causes of the disease and to establish diagnostic strategies [14]. These studies have highlighted differences in amino acids, lipids, and imbalances in energy and redox metabolisms. However, it is important to note that no consistently altered metabolites were identified in all studies, which poses a challenge to a full understanding of the disease.

The surprising nature of ME/CFS, in which multiple organ systems are affected [6,7,8,9,10,11,12,13,14], underlines the complexity of the disease and the need for further research. ME/CFS is a heterogeneous condition, and individual variations in symptoms and underlying mechanisms may contribute to the difficulty in identifying consistent biomarkers or metabolic changes. More comprehensive and collaborative research efforts are required to uncover the mechanisms underlying ME/CFS, identify reliable biomarkers, and develop targeted therapies. The involvement of multiple organ systems highlights the importance of a multidisciplinary approach in the diagnosis, treatment, and management of this complex disease.

Explainable artificial intelligence (XAI) based on machine learning has recently been utilized in healthcare diagnostics [4,15]. Valdes et al. applied an XGBoost model to predict the prevalence, demographics, and costs of ME/CFS. The model was developed based on the characteristics of individuals diagnosed with ME. The results showed a prevalence rate of 835/100,000 in the United States population study [4]. Yagin et al. proposed an XAI model to extract gene biomarkers for COVID-19. The model applied local interpretable model-agnostic explanations (LIME) and SHAPley Additive exPlanations (SHAP) approaches, which identified three genes that can predict the disease with an accuracy of 93% [15].

In this study, we comprehensively analyzed the metabolites of ME/CFS patients compared to normal controls to identify patterns in metabolites that could potentially serve as biomarkers for the disease. What makes our analysis comprehensive is that we examined metabolites belonging to nine different super pathways, aiming to address the heterogeneous nature of the disease and understand its mechanisms of development and progression. To achieve this, we used a combination of XAI methodology and ML. This methodology enabled us to identify discriminative metabolites for ME/CFS.

## 2. Materials and Methods

### 2.1. ME/CFS Metabolomics Dataset

The metabolomics data of CFS patients and healthy controls were utilized to perform the experiments in the study [2]. All of the participants were female and consisted of 26 healthy controls and 26 ME/CFS patients aged 22 to 72 years and with a similar body mass index (BMI). Data for 768 different identified metabolites were obtained from the plasma sample used in the global metabolomics panel. According to the standards set by Metabolon^®^, the detected substances were further classified into nine different metabolic super-pathways. The distribution of identified compounds is as follows: amino acids 196, carbohydrates 25, cofactors and vitamins 29, energy 10, lipids 259, nucleotides 33, partially defined molecules 2, peptides 33, and xenobiotics 181 (Supplementary Files S1 and S2).

### 2.2. Experimental Setup and Proposed Framework

**Figure 1:**
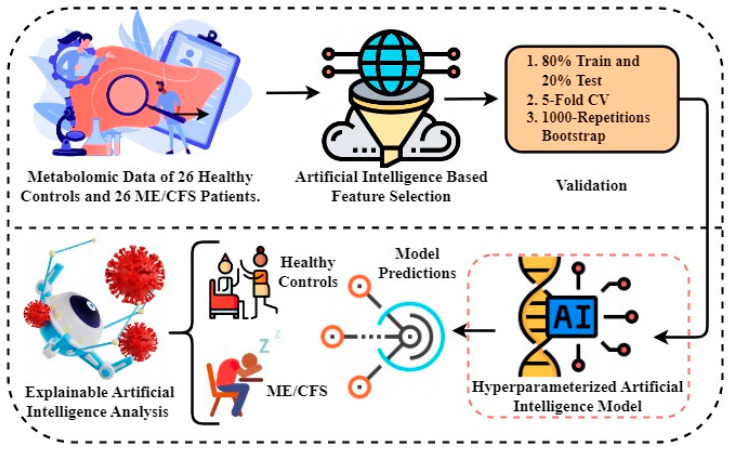
The proposed methodology architecture analysis for detecting healthy individuals and ME/CFS patients.

The Python programming language was used to perform the research experiments. The experiments were conducted in an environment containing a graphics processing unit (GPU) backend with 16 GB of RAM and 90 GB of disk space. sklearn version 1.2.2, numpy version 1.22.4, seaborn version 0.12.2, pandas version 1.5.3, and matplotlib version 3.7.1 were the used machine learning libraries. An architectural representation of the proposed methodology is depicted in Figure 1. Diagnosis and biomarker discovery of patients suffering from ME/CFS and healthy controls form the basis of this proposed study. Below is a step-by-step description of the proposed methodology:

- The first step involves obtaining metabolomics data to be used in the experiments. Metabolomics data are based on results from a study of 26 healthy controls and 26 ME/CFS patients aged 22 to 72 years with similar BMI.
- In the second step, artificial intelligence-based random forest (RF) feature selection is applied to identify biomarker candidate metabolites and to eliminate the high dimensionality problem in omics data. Because the metabolomics data have a large number of feature dimensions, the performance scores of the predicted models may be lower. Therefore, the twenty most important metabolites contributing to improved performance scores in ME/CFS prediction were identified.
- In the third step, 80–20% split, 5-fold cross-validation (CV), and 1000 replicate bootstrap approaches were used to validate the prediction models to be generated using the selected biomarker candidate metabolites, and the results were compared.
- In the fourth step, Bayesian hyper-parameter optimization was used to determine the optimal parameters.
- In the fifth step, predictive models were built to diagnose ME/CFS patients. For this purpose, the Gaussian naive Bayes (GNB), gradient boosting classifier (GBC), logistic regression (LR), and random forest classifier (RFC) algorithms were constructed. The performance of the models was evaluated using the area under (AUC) receiver operating characteristic (ROC) curve, the Brier score, accuracy, precision, recall, and the F1 score. While the primary purpose of the methodology is biomarker discovery and diagnosis of ME/CFS, an important secondary purpose is to provide users with indicative probability scores. Therefore, we evaluated the quality of the probabilities with a calibration curve and by calculating the Brier score.
- Finally, XAI approaches SHAP and TreeMap were applied to the proposed model in order to provide transparency and interpretability to the model and to explain intuitively the decisions made by the model. With the help of SHAP and TreeMap, the rationale and process behind a particular decision made by the proposed model can be grasped.

### 2.3. Feature Selection

For feature selection and dimensional reduction from the utilized metabolomics data, the RF method, which is based on artificial intelligence, was applied in this study. The mean decrease impurity method is commonly utilized to carry out the process of choosing features that are included in the random forest model. The impurity of the decision trees in the forest is used as a factor in the calculation of the important score for each feature. This score is based on the average amount that each feature reduces the impurity of the decision trees. The feature importance score was normalized in such a way that the total of all features’ importance values is equal to 1. After that, the most important features with the highest scores are chosen to be used for training the models that are applied. The RF method of feature selection can be mathematically represented as follows:

$$\begin{array}{l} Feature\;Importance\\ \;\;\;\;\;\;\;\;\;\;\;\;\;\;\;\;\;\;=\frac{1}{n_{trees}}*\sum_{t=1}^{n_{trees}}\sum_{t=1}^{n_{nodes}}I_{v_{i}=f}*\left(\begin{array}{c} N_{t}\\ N \end{array}\right)\\ \;\;\;\;\;\;\;\;\;\;\;\;\;\;\;\;\;\;\;*\left({impurity}_{parent}-{impurity}_{childern}\right) \end{array}$$

where:

*n_trees_* is the number of decision trees in the random forest.

*v_i_* is the feature used for the split at node *i* of the *t*-th tree.

*f* is the feature being evaluated for importance.

*I_vi_* = *f* is an indicator function that equals 1 if *v_i_* = *f* and 0 otherwise.

*N_t_* is the number of samples in the *t*-th tree that reaches node *i*.

*N* is the total number of samples in the training set.

Impurity parent is the impurity of the set of samples at the parent node *i*.

Impurity child is the weighted impurity of the two sets of samples after the split based on feature *f*.

### 2.4. Validation Methods

The evaluation of a predictive model is prone to overestimation when assessed only on the population of individuals that was utilized to construct the model [16]. There exist various internal validation methods that strive to offer a more precise estimation of model performance on novel subjects [17,18]. We conducted an evaluation of multiple variations of holdout, cross-validation [17], and bootstrapping [17] methodologies. These validation methods were used to evaluate the distinctive performance and calibration quality of our ML methodology on metabolomics ME/CFS data.

**Hold-out Validation:** One commonly used and well-accepted methodology involves randomly partitioning the training dataset into two distinct subsets: one for model development and the other for evaluating its performance. The performance of the model is evaluated using a split-sample (hold-out) approach, where identical yet independent data are utilized. The hold-out validation approach involves utilizing the training data samples during the training phase, while the testing data samples are utilized to assess the predictive performance of the model [19].

**k-fold Cross-validation:** A more advanced technique involves the utilization of cross-validation, which can be regarded as an expansion of the split-sample methodology [16]. Split-half cross-validation involves developing a model using one randomly selected half of the data and subsequently testing it on the other half, and vice versa. The average is commonly used as a means for approximating performance. Certain percentages of respondents may be omitted, such as 10%, in order to evaluate a model that was constructed using 90% of the sample. The aforementioned technique is iterated a total of ten times, ensuring that each subject is tested once to evaluate the model. k-fold cross-validation is a statistical method for evaluating and comparing learning algorithms; in k-fold cross-validation, the data are first divided into k folds, each of which has a size that is equal to or very close to being equal to the others. Following this, k iterations of training and validation are carried out in such a way that, within each iteration, a different fold of the data is held out for validation while the remaining k minus one folds are used for learning [20].

**Bootstrap Validation:** It has been argued that computationally demanding resampling methods like the bootstrap technique provide the most reliable validation [16]. The bootstrapping technique involves generating samples from a given population by randomly selecting samples with replacement from the initial dataset, where the size of the generated samples matches the size of the original dataset.

The estimation of prediction error using cross-validation is generally unbiased, although it can exhibit significant variability. However, the bootstrap method produces findings with low variance. Bootstrap validation can be conceptualized as a technique that generates smoother versions of cross-validation. Bradley and Robert demonstrated that the bootstrap method exhibited superior performance compared with cross-validation in a collection of 24 simulation experiments [17]. The bootstrap resampling method is a way to predict the fit of a model to a hypothetical test set when an explicit test set is not available [21]. It helps to avoid overfitting and improves the stability of ML algorithms [22].

### 2.5. The Bayesian Approach for Hyper-Parameter Optimization

The effectiveness of an ML model is determined by the hyper-parameters associated with that model. Hyper-parameters have influence over the learning process or the structure of the statistical model that lies beneath the surface. On the other hand, there is no standard approach to selecting hyper-parameters in real experiments. As a substitute, practitioners frequently set hyper-parameters using a process of trial and error or occasionally allow them to remain at their default settings, both of which result in inadequate generalization. By recasting it as an optimization problem, hyper-parameter optimization gives a methodical approach to solving this issue. According to this line of thinking, a good set of hyper-parameters should (at the very least) minimize validation errors. When compared with the vast majority of other optimization problems that can arise in machine learning, hyper-parameter optimization is a nested problem. This means that at each iteration, an ML model needs to be trained and validated. Many approaches have been developed to discover the optimal combination of ML model hyper-parameters. Grid search and random search are two optimization approaches that are often used for this purpose. These strategies, however, have a few drawbacks. Grid searching is a time-consuming and inefficient strategy for the central processing unit (CPU) and graphics processing unit (GPU). The grid search strategy outperforms random search; nevertheless, the exact answer is more likely to be ignored. In comparison to these two strategies, Bayesian optimization is the best choice for searching for hyper-parameters. First, because the Gaussian process is involved, the Bayesian optimization technique may consider prior results. To put it another way, each step computation may be retrieved to assist in determining a better set of hyper-parameters. Second, compared with other methodologies (for example, grid search), Bayesian optimization takes fewer iterations and has a quicker processing time. Finally, even when working with non-convex issues, Bayesian optimization may be trusted [23,24,25,26].

### 2.6. Classification Models

To separate patients into two categories, namely, ME/CFS and healthy individuals, we made use of a variety of AI-based classification algorithms in this work. These include GNB, GBC, LR, and RFC.

**GNB:** The GNB algorithm is a well-known classification method that is frequently utilized in the field of biomedical research to categorize various patient groups. GNB can be used to properly diagnose patients based on specific physiological traits or biomarkers in the case of healthy individuals as well as patients with ME/CFS. The Bayes theorem, which asserts that the probability of a hypothesis may be computed based on the probability of observing specific evidence, serves as the foundation for GNB’s mathematical operation. This theorem underpins how GNB works. When using GNB, it is assumed that the conditional probability of each feature given the class is Gaussian, which indicates that the features are regularly distributed within each class. This enables compliance with the requirements of the GNB algorithm. This assumption makes the computation of the posterior probability much easier, which in turn enables classification that is both more efficient and more accurate. The GNB method works by first determining the posterior probability of each class for a certain set of features and then designating the class that has the highest probability as the class that will be predicted [27,28]. The following mathematical notations are used to express GNB:

$$P\left(x|y\right)=P\left(x|y\right)\times \frac{P(y)}{P(x)}$$

where:

*P*(*y*|*x*) is the posterior probability of class *y* given input vector *x*.

*P*(*x*|*y*) is the likelihood of the input vector *x* given class *y*, modeled as a multivariate Gaussian distribution.

*P*(*y*) is the prior probability of class *y*, estimated as the relative frequency of *y* in the training set.

*P*(*x*) is the evidence or marginal likelihood of the input vector *x*, calculated as the sum of the joint probabilities of *x* and all possible classes *y*.

**GBC:** The GBC is a powerful ML algorithm that has shown great potential in the classification of healthy individuals and patients with ME/CFS [29]. The GBC operates by iteratively constructing an ensemble of weak prediction models, typically decision trees, and combining their outputs to make accurate predictions. During the training phase, the GBC builds the ensemble by initially fitting a weak model to the training data. Subsequent models are then constructed in a way that each new model focuses on the instances that were previously misclassified by the ensemble [30,31]. The mathematical notations for the GBC model for classification are as follows:

$$\hat{y_{i}}=\sum_{M=1}^{M}f_{m}(x_{i})$$

where:

$\hat{y_{i}}$ represents the predicted value for the *i*-th instance.

*M* denotes the number of weak classifiers (decision trees) used in the GBC.

$f_{m}(x_{i})$ refers to the *m*-th weak classifier’s prediction for the *i*-th instance.

**LR:** For binary classification problems such as disease categorization using patient data, LR is a common machine learning model. Given input data including patient demographics, symptoms, and laboratory test results, a LR model calculates the likelihood of a positive class. To maximize the likelihood of the positive class, the model learns the ideal set of weights or coefficients by minimizing the logistic loss function. To produce a probability between 0 and 1, the logistic function uses a linear combination of input features and their weights. Afterward, a threshold (such as 0.5) is used to the anticipated probability to determine the expected class; if the predicted probability is greater than the threshold, the positive class is predicted, and vice versa [32,33]. The following are some mathematical symbols for the LR model of binary classification:

$$p\left(y=1|x,\theta \right)=\frac{1}{\left(1+e^{-(\theta_{0}+\theta_{x_{1}}+\theta_{x_{2}}+\cdots +\theta_{xp})}\right)}$$

where:

*p*(*y* = 1|*x*,*θ*) is the predicted probability of the positive class given the input feature vector *x* and the model parameters *θ*.

*e* is the base of the natural logarithm (approximately 2.718).

*θ*_0_ is the intercept or bias term.

*θx*_1_, *θx*_2_, …, *θx*_0_ are the coefficients or weights of the input features *x*_1_, *x*_2_, …, *x_p_*.

*x* = [*x*_1_, *x*_2_, …, *x_p_*] is the input feature vector.

**RFC:** RFC is a well-known technique for machine learning that is used for classification tasks, such as the classification of diseases based on patient data. Building an ensemble of decision trees that have been trained on random subsets of the input features and data samples is how RFC goes about completing its work. Each decision tree in the ensemble makes a prediction based on a subset of the input features, and the final prediction is generated by aggregating the predictions of all of the trees in the ensemble. RFC can handle high-dimensional data with a large number of features and can also capture nonlinear correlations between the input features and the output classes [34,35,36]. The RFC equation is written as follows in its mathematical notation:

y=f(X)

where:

*X* is an input data matrix with *n* samples and p features, where *X* = [*x*_1_, *x*_2_, …, *x_n_*] and each *x_i_* is a vector of p features.

*y* is a vector of predicted class labels, where *y* = [*y*_1, *y*_2, …, *y*_*n*].

*f*(*X*) is the function that maps the input data *X* to the predicted class labels *y* using a random forest model.

### 2.7. Performance Evaluation and Model Calibration

#### Performance Evaluation

**Accuracy:** Accuracy refers to the correct classification rate of a classification model. The accuracy score is calculated as the ratio of correctly guessed samples to the total number of samples. However, in the case of unbalanced classes or misclassification costs, the accuracy score alone may be insufficient and should be evaluated in conjunction with other metrics [37].

**Precision:** The precision score expresses how many of the positively predicted samples are actually positive. The precision score is calculated as the ratio of the number of false positives (False Positive) to the total number of positive predictions (True Positive + False Positive). The higher the precision score, the better the positive predictions of the model [37].

**Recall:** The recall score expresses how many of the true positives (True Positive) are correctly estimated. The recall score is calculated as the ratio of the number of false negatives (False Negative) to the total number of true positives (True Positive + False Negative). The higher the recall score, the better the model captures true positives [37].

**F1 Score:** The F1 score is calculated by taking the harmonic mean of the precision and recall scores. It is preferred to the harmonic mean because it provides the balance between precision and recall scores. The higher the F1 score, the higher the model classifies with both high precision and high recall [37].

**ROC Curve and AUC:** The evaluation of diagnostic tests is a topic of interest in contemporary medicine, and this is true not only for determining whether or not a disease is present in a patient but also for determining whether or not healthy people have the disease. The conventional method of diagnostic test evaluation uses sensitivity and specificity as measures of accuracy of the test in comparison with the gold standard status. This method is used in diagnostic tests that have a binary outcome, such as positive or negative results from the test. In a scenario in which the test results are recorded on an ordinal scale (for example, a five-point ordinal scale: “definitely normal”, “probably normal”, “uncertain”, “probably abnormal”, and “definitely abnormal”), or in a scenario in which the test results are reported on a continuous scale, the sensitivity and specificity can be computed across all the possible threshold values. Therefore, the sensitivity and specificity change throughout the different thresholds, and there is an inverse relationship between sensitivity and specificity. Then, the receiver operating characteristic (ROC) curve is called the plot of sensitivity versus 1-Specifity, and the area under the curve (AUC) is a reliable indicator of accuracy that has been considered with relevant interpretations. ROC curves are plotted as sensitivity versus 1-Specifity. This curve is extremely important when determining how well a test can differentiate between different types of people and their actual conditions. A ROC curve is formed when sensitivity vs specificity is plotted against each other across a range of cutoffs. This plot forms a curve in the unit square. In “ROC space”, the ROC curves that correspond to diagnostic tests with progressively stronger discriminant capacity are situated gradually closer to the upper left-hand corner. The area under the curve is a statistic that provides a comprehensive overview of the ROC curve rather than focusing on a single point of operation. AUC represents the area under the ROC curve and takes a value between 0 and 1. The AUC value measures the discrimination ability of the classification model. A high AUC value means that the model can discriminate well and has a high sensitivity and low false positive rate. The closer the AUC value is to 1, the better the model’s performance [38,39,40].

### 2.8. Model Calibration

A well-calibrated model is one in which the estimated probability matches the true incidence of the outcome. For example, approximately 90% of patients with an estimated risk of ME/CFS of 0.9 would be classified as ME/CFS. This is critical for prediction models because clinical decision-makers need to know how confident the model is in making a particular prediction. Therefore, we calibrate the trained model to obtain the correctly predicted probability. In this article, we use the Brier score and calibration curve for model calibration [41,42].

**Brier Score:** The Brier score is a metric used to evaluate the quality of probability estimates. It is especially used for probabilistic classification models. The Brier score provides a measure of the mean squared errors between the actual labels and the estimated probabilities. The lower the Brier score, the closer the predictions are to reality [41,42].

**Calibration Curve:** A calibration curve is a tool used to evaluate how close a classification model’s estimates are to the true probabilities. This curve shows the accuracy of the probabilities predicted with the model. The calibration curve is important to determine the confidence level of the model and to evaluate the reliability of the predictions. A well-calibrated model means that high-probability predictions are more likely to happen, while low-probability predictions should be less likely to happen. It has been verified that the probabilities predicted by a well-calibrated model are consistent with the realization rates [41,42].

### 2.9. XAI Approaches

Interpretability is absolutely essential when using a complex ML model in a real-world environment such as the medical field. XAI is an emerging research area that aims to increase the interpretability and transparency of applied ML models. XAI ensures that decisions made with applied models are understood and trusted, especially in critical applications such as healthcare. XAI techniques can help users understand, validate, and trust the decisions made with these models in real-world applications [43,44]. In this research, Shapley values and the TreeMap approach were used to interpret the estimation decision of the optimal ML model.

**Shapley Additive Explanations** (**SHAP**)**:** This is an approach used to understand the contribution of each feature to the prediction to explain the predictions of SHAP ML models. This approach also takes into account the complexity of the ML model and the interactions between features that go into the model. It also measures the contribution of a feature to the prediction using Shapley values and thus produces graphical results for understanding the model’s decisions [44].

**TreeMap:** TreeMap provides an intuitive description for tree-based ML models, showing the name of the property used for each decision level and the split value for the condition. If an instance satisfies the condition, it goes to the left branch of the tree; otherwise, it goes to the right branch. When purity is high in TreeMap, the knuckle/leaf has a darker color. The samples row at each node shows the number of samples examined at that node [45].

## 3. Results

In this section, firstly, the results of biomarker candidate metabolites are given, and the performance results of the applied predictive artificial intelligence algorithms are evaluated using various evaluation metrics. Predictive models were constructed based on both the original data and the metabolites identified as biomarker candidates, and the results were compared. The model showing the final performance was used for ME/CFS estimation, and we tried to explain the decision-making function of the model explained using XAI approaches.

### 3.1. Feature Selection Results

**Figure 2:**
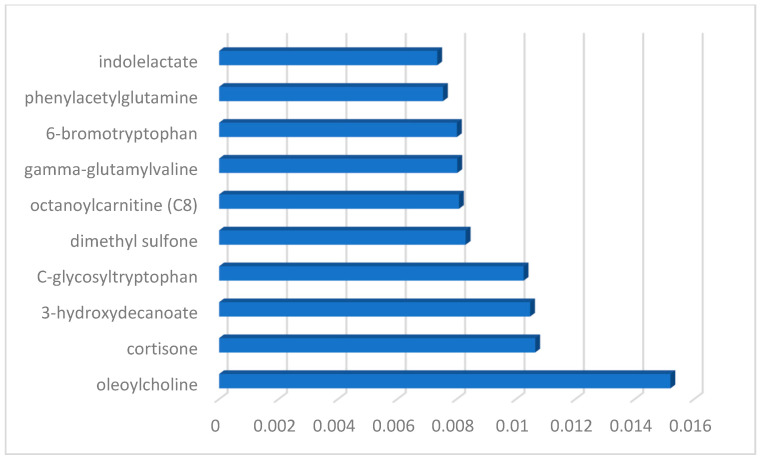
The histogram-based feature importance plot of selected features using the RF model.

Figure 2 depicts the features that were selected together with their respective relevance ratings, which were determined using a random forest method based on machine learning. The results reveal that the metabolomics of oleoylcholine, cortisone, 3-hydroxydecanoate, and C-glycosyltryptophan are extremely relevant for the diagnosis of ME/CFS patients.

### 3.2. Hyper-Parameters Optimization Results

In Table 1, the optimal hyper-parameters of ML models according to Bayesian optimization are given.

### 3.3. The Model Performance Results

In this section, we show how selecting metabolic traits associated with ME/CFS can help learning models improve their performance. After training using all input features and a subset of them (significant features), the results of all used models (GNB, GBC, LR, RFC) are presented in Table 2. We also performed three experiments for model validation: in the first experiment, the dataset was split into 80% and 20% to train and validate the learning models. In the second experiment, we used the cross-validation method during the training and validation of the learning models. Finally, we used the 1000-iteration bootstrap method in our last experiment. Bootstrap is a resampling method in which parts are changed at each iteration of the sampling process. This creates a randomly selected collection of samples from the set of input samples. This procedure can be performed k times. Models were trained on the sampled dataset and evaluated on the original dataset. In all experiments, we calculated the performance of all learning models with and without a feature selection step. After the three experiments outlined in this section, the results of each learning model were accuracy (A), precision (P), recall (R), F1 score (F1), Brier score (B), and AUC.

According to Table 2, for the models using the original features in the dataset, it was determined that the GBC model achieved the lowest performance scores (accuracy: 36%; AUC: 33%; Brier score: 0.63) when dividing the data into 80–20. Based on the result of bootstrap validation with the original features, the LR model had the best performance (accuracy: 96%; AUC: 95%; Brier score: 0.04). All results for the models using biomarker candidate metabolites showed improved prediction performance when compared with the models using the original features. The results of the investigation show that the performance metrics scores of all the used machine learning approaches for diagnosing healthy controls and ME/CFS patients were much improved by applying selected biomarker metabolites. The interpretation was also more likely for these models, which took into account fewer risk factors. The bootstrap validation method gave superior results compared with the first two experiments (80–20 split and five-fold CV) both in experiments using the original metabolomics variables and in models using twenty biomarker candidate metabolites. It was determined that the RFC learning model outperformed the other three models (GNB, GBC, and LR) with the 1000-iteration bootstrapping method for ME/CFS prediction based on a few metabolite markers. The RFC learning model achieved 98% accuracy, 98% precision, 98% recall, 98% F1 score, 0.01 Brier score, and 99% AUC.

**Figure 3:**
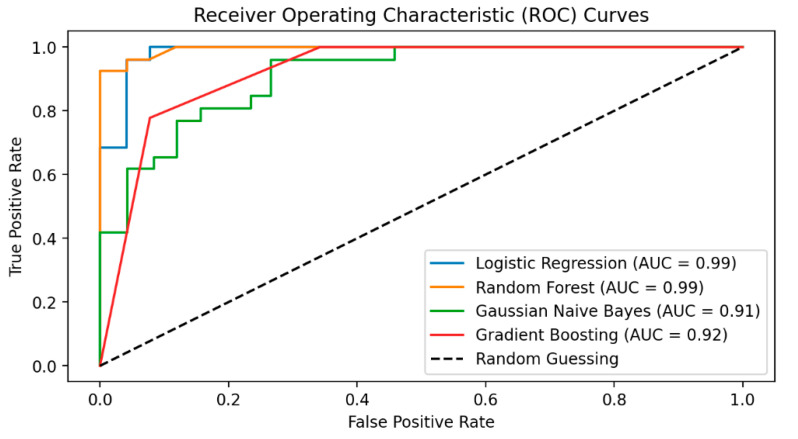
The attained AUC of all ML models after being trained/validated using the biomarker metabolites.

The ROC area reached by each learning model after training on the selected biomarkers is shown in Figure 3. The better the performance of the prediction model, as measured using the ROC curve, the closer the value of the AUC is to one. As can be seen in Figure 3, the RFC model reached its highest AUC value of 99%.

**Figure 4:**
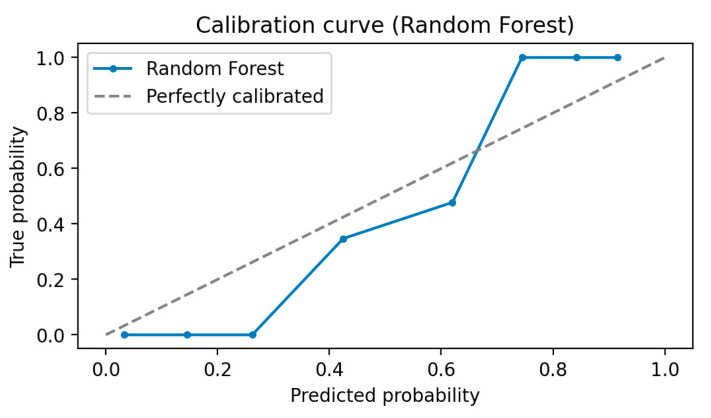
The calibration curve analysis of the outperformed RFC model.

It is common in classification to seek both an estimate of the class label and the probability of that label. By examining these possibilities, the diagnostic decision of the learning model can be more relied upon. A well-calibrated model is one in which the estimated probability matches the true incidence of the outcome. For example, approximately 90% of patients with an estimated risk of ME/CFS of 0.9 will develop ME/CFS. This is critical for models because clinical decision-makers need to know how confident a model is in making a particular prediction. Therefore, we plotted the calibration curve for the trained model in Figure 4 to obtain the correctly predicted probability. To calibrate the accuracy of the estimates, the calibration process compares the actual tag frequency with the expected tag probability. A closer alignment of points along the major diagonal of the graph indicates a more accurate calibration or a more reliable estimate. The calibration curve showed a good fit of the model (Figure 4).

### 3.4. XAI Results

**Figure 5:**
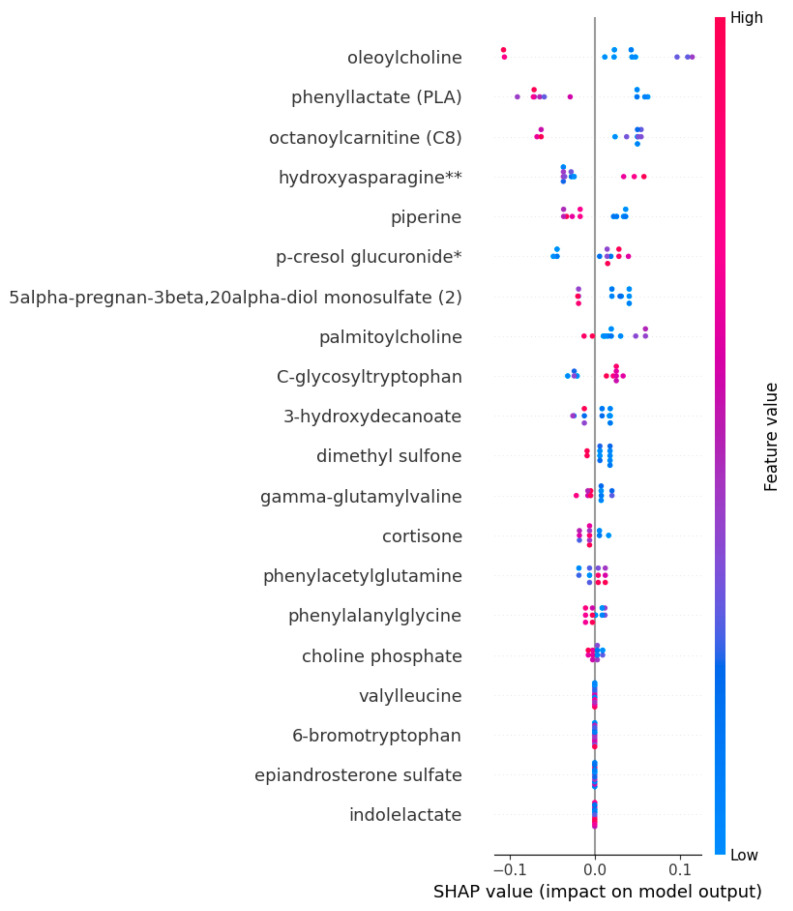
The explainable impact of the proposed RFC model output on biomarker metabolite features.

SHAP was used to identify metabolomics biomarkers according to their importance or contribution to the prediction of ME/CFS and to explain the prediction decisions of the model. The RFC-trained model was subjected to SHAP annotation, which identified the most important trait metabolites responsible for the prediction of ME/CFS. The results pointed to a list of metabolites with importance scores. Metabolite biomarkers are arranged in decreasing order of importance. Oleoylcholine, phenylactate (PLA), octanoylcarnitine (CB), hydroxyasparagine**, and piperine are among the most prominent metabolite biomarkers important in the diagnosis of ME/CFS. Figure 5 also visualizes the relationships between the relative value of biomarker candidate metabolites and the SHAP values for these metabolites. In each row of the graph, each patient is marked as a dot. The horizontal position of the dot reflects the SHAP values, and the color of the dot encodes the relative value of the metabolites and their mean in the dataset. A positive SHAP value denotes a positive contribution to the target variable, whereas a negative SHAP value denotes a negative contribution.

Therefore, low values (relatively blue) of the oleoylcholine and phenylactate (PLA) metabolites contribute positively to ME/CFS, thus increasing disease risk. In addition, it was determined that high levels of hydroxyasparagine**, p-cresol glucuronide*, and C-glycosyltryptophan metabolites increased the risk of ME/CFS (Figure 5).

**Figure 6:**
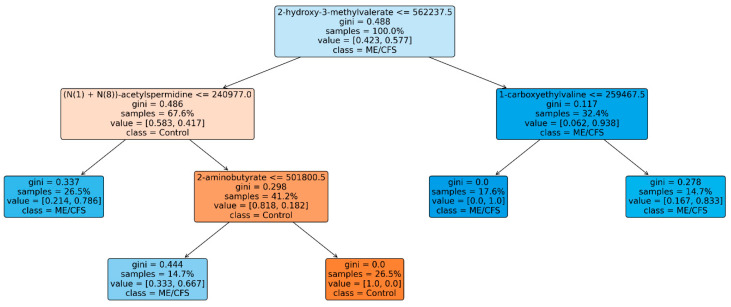
The TreeMap space analysis of the proposed RFC model.

In addition to that, to gain an understanding of how the RFC model behaves, we used a method that is known as TreeMap analysis. Figure 6 is an illustration of the TreeMap that is included in the RFC. The analysis explains how the proposed model came to its conclusion about the classification of patients as healthy controls and those with ME/CFS.

## 4. Discussion

Fatigue is a common occurrence in human beings and serves as an indicator of disrupted homeostasis within the body, resulting from either excessive physical and mental exertion or illness [46]. In addition to being one of the most significant social concerns, chronic fatigue additionally constitutes the most significant economic losses [47]. Pain, cognitive dysfunction, autonomic dysfunction, sleep disturbance, and neuroendocrine and immune symptoms are just some of the many symptoms associated with ME/CFS [48]. A patient must have a symptom of neurological impairments, an immune/gastrointestinal/genitourinary impairment, and an energy metabolism/transport impairment to be diagnosed with ME/CFS and meet the criteria for post-exertional neuroimmune exhaustion. However, the strength and severity of such symptoms in a patient vary and are heterogeneous, from moderate to severe, with some patients even becoming bed-bound [48]. Because it is challenging to identify the typical abnormal elements for this disorder utilizing general and conventional medical examination, artificial intelligence-based automated methods may aid in improving the diagnosis of ME/CFS. In recent years, a growing number of studies have explained the pathology of ME/CFS and have established biomarkers for the same by using a metabolome analysis technique [48,49,50]. This has allowed for the development of a variety of diagnostic studies [51,52].

The present study was an investigation of the effectiveness of the methodology combining ML and XAI techniques to investigate biomarkers of ME/CFS and develop an interpretable predictive model for disease diagnosis. Metabolomics data from patients diagnosed with ME/CFS and healthy controls were used. The classification algorithms included GNB, GBC, LR, and RFC. The classifiers’ performance was evaluated both with and without the implementation of the feature selection algorithm (RF). In addition to classical hold-out validation, cross-validation and bootstrap approaches were also used to evaluate the performance of the classification learning algorithms in the validation stage, and the effectiveness of these three validation approaches was also examined. Shapely values, an explainable AI system, were utilized to interpret the classification models’ predictions and decisions. After being trained and validated on the significant selected features using the bootstrapping method, the RFC model was found to be superior to the other four models (GNB, GBC, and LR). Accuracy, precision, recall, F1 Score, and the AUC were all at or above 98% for the RFC model. The higher the values attained for precision and sensitivity, the higher the proportion of correct diagnoses, also known as true positives (TPs), and the lower the value of false negatives (FNs). Errors, both positive and negative, known as false positives (FPs) and FNs, are widespread in comparative biology research. In addition, we demonstrated that our method was capable of demonstrating the main features as well as the interpretations of ML findings by utilizing SHAPley values and SHAP plots. The SHAP method’s findings indicated that oleoylcholine, phenyllactate, octanoylcarnitine, hydrooxyasparagine, piperine, p-cresol glucuronide, and palmitoylcholine are all chemicals associated with ME/CFS and crucial to the model’s final decision. The use of the SHAP technique revealed that the indolelactate, which has low Shapley values, is the least significant of all the features. On the other hand, the feature with the highest Shapley value is oleoylcholine, which is also the one that contributes the most significant information for the diagnosis of ME/CFS. Oleoylcholine is a member of the class of chemical compounds known as acylcholines. Germain et al. [2] researched the metabolic pathways that influence the diagnosis of ME/CFS patients by performing statistical analysis in conjunction with pathway enrichment analysis. They found that acylcholines, which are part of the sub-pathway of lipid metabolism, known as fatty acid metabolism, are consistently reduced in two different patient cohorts that suffer from ME/CFS. Nagy-Szakal et al. [53] gained insights into ME/CFS phenotypes using comprehensive metabolomics. Biomarker identification and topological analysis of plasma metabolomics data were performed on a sample group consisting of fifty ME/CFS patients and fifty healthy controls. They demonstrated that patients with ME/CFS have higher plasma levels of ceramide and observed that there is a variation in the level of carnitine, choline, and complex lipid metabolites. The study of plasma metabolomics data attained a more accurate prediction model of ME/CFS (AUC = 0.836).

A comprehensive metabolomics analysis was conducted by Naviaux et al. [54] to better understand the biology of CFS. They investigated 612 plasma metabolites across 63 different metabolic pathways. Twenty metabolic pathways were revealed to be abnormal in patients with chronic fatigue syndrome. Sphingolipid, phospholipid, purine, cholesterol, microbiota, pyrroline-5-carboxylate, riboflavin, branch chain amino acid, peroxisomal, and mitochondrial pathways were all disrupted. Diagnostic accuracies of 94% were found using AUC characteristic curve analysis. In our experiment utilizing the ML-based model, we were able to achieve a greater level of accuracy (98%) for our proposed prediction model: the RFC model. Petrick and Shomron [55] discussed how well an ML-based model performs. They highlighted how AI and ML have permitted important breakthroughs in untargeted metabolomics workflows and key findings in the fields of disease diagnosis. In conclusion, the proposed model (RFC) was successful in correctly diagnosing ME/FCS patients. The findings indicate that ML, when paired with the Shapely analysis, is able to explain the ME/FCS classification model and offer physicians basic knowledge of the main metabolic chemicals that influence the model decision. Clinicians can benefit from individual explanations of the important metabolic compounds in order to gain a better grasp of why the model yields certain diagnoses for individuals with ME/CFS.

In the context of classification, it is crucial to accurately estimate the true error rate of a specific classifier in certain situations. CV is a conventional methodology that is almost unbiased but exhibits a high degree of variability. The bootstrap method is an alternative strategy that provides a more stable testing for small sample sizes. The bootstrap method is generally acknowledged to exhibit superior performance for small sample sizes due to its reduced variance [17]. The aforementioned rationale prompted us to explore alternative methodologies, such as hold-out validation and CV, in this study. This decision was made due to the limited sample size of our medical application, which consisted of 26 healthy controls and 26 ME/CFS patients in the current study.

In the experiments, we conducted a comprehensive evaluation of diverse validation techniques (such as hold-out, bootstrap, and k-fold CV) across a range of models using metabolomics ME/CFS data. A relevant prior study [56] emphasized the importance of prioritizing repeated CV and bootstrap methodologies for studies with limited sample sizes, as opposed to relying solely on a hold-out approach or a small external test set with similar patient characteristics. In a study comparing CV and bootstrap results in the literature, the authors reported that both resampling techniques are effective, but in some cases, the bootstrap resampling technique is better [57]. While it was highlighted that repeated CV with a complete training dataset emerged as a preferred choice [56], our specific findings demonstrated that the bootstrap validation method, involving 1000 repetitions, yielded the highest classification outcomes. Given the insights from our current investigation, it is plausible to recommend a holistic approach, where ML models are coupled with various validation methods, thereby selecting the algorithm that exhibits the optimal performance. This underscores the significance of tailoring validation techniques to a specific dataset and context, rather than adhering rigidly to a single method. By embracing this approach, researchers can harness the synergy between ML and versatile validation strategies, leading to enhanced model reliability and predictive accuracy.

## 5. Conclusions

Although research into the causes and mechanisms of ME/CFS continues, the exact underlying factors are not yet fully understood. It has been reported to result from a complex interaction of biological, genetic, environmental, and psychological factors. Advances in research are crucial for better understanding the disease, improving diagnosis and treatment options, and ultimately finding a cure. Based on this information, the RFC model proposed in this study correctly classified and evaluated ME/CFS patients using the selected biomarker candidate metabolites. The methodology combining ML and XAI can provide a clear interpretation of risk estimation for ME/CFS, helping physicians intuitively understand the impact of key metabolomics features in the model.

## 6. Limitations and Future Works

This study lacked a third-party verification by an independent biologist, which may have provided more explanation of the collected results, vital metabolic chemicals, and their significance to the diagnosis of patients with ME/FCS. It is vital to broaden the present investigation further by incorporating multicenter experiments in subsequent research or to make use of the associated data from multiple locations for external validation. The size of the metabolomics dataset might be increased by collecting additional samples from patients. This would be an improvement for this line of investigation. The performance of patient diagnosis can be improved with the development of advanced transfer learning-based methodologies.

## Figures and Tables

**Table 1:** The hyper-parameter tuning analysis of applied methods.

Technique	Optimized Parameter Value
GNB	var_smoothing = 1 × 10^−9^
GBC	n_estimators = 3, learning_rate = 1.0, max_depth = 1
LR	max_iter = 30, solver = ‘liblinear’
RFC	max_depth = 26, min_samples_leaf = 5, min_samples_split = 3, n_estimators= 12

**Table 2:** The comparative performance analysis of the applied artificial intelligence techniques with different approaches.

Attained Performance Using All Input Features							Attained Performance Using Feature Selection					
Technique	A (%)	P (%)	R (%)	F1 (%)	B	AUC (%)	A (%)	P (%)	R (%)	F1 (%)	B	AUC (%)
	80–20% Split Validation						80–20% Split Validation					
GNB	73	72	73	72	0.27	67	73	72	73	72	0.27	67
GBC	36	39	36	37	0.63	33	73	75	73	73	0.27	73
LR	64	64	64	64	0.36	60	73	72	73	72	0.27	67
RFC	45	56	45	44	0.54	51	82	86	82	80	0.18	75
**Results with a 5-fold cross-validation**							**Results with a 5-fold cross-validation**					
GNB	52	36	94	62	0.26	59	82	77	92	84	0.15	91
GBC	48	47	35	37	0.34	52	95	94	99	95	0.05	98
LR	58	46	71	54	0.45	46	95	95	96	96	0.03	98
RFC	56	68	38	56	0.28	64	97	96	97	98	0.04	99
**Results with a 1000-repetition bootstrap**							**Results with a 1000-repetition bootstrap**					
GNB	63	70	63	60	0.36	63	83	84	83	83	0.17	91
GBC	92	92	92	92	0.07	92	96	96	96	96	0.03	92
**LR**	**96**	**96**	**96**	**96**	**0.04**	**95**	96	96	96	96	0.04	99
**RFC**	90	90	90	90	0.09	90	**98**	**98**	**98**	**98**	**0.01**	**99**

GNB: Gaussian Naïve Bayes; GBC: gradient boosting classifier; LR: logistic regression; RFC: random forest classifier; A: accuracy; P: precision; R: recall; B: Brier score; AUC: area under the ROC curve.

## Data Availability

The dataset used in this study is provided as a link in the Supplementary Materials of this article.

## Supplementary Materials

The following supporting information can be downloaded at: https://www.mdpi.com/article/10.3390/diagnostics13233495/s1, Supplementary File S1: OrigScale HD4; Supplementary File S2: OrigScale CLP.
